# Neutrophil-to-Lymphocyte Ratio and Thrombocyte-to-Lymphocyte Ratio as a Predictor of Severe and Moderate/Mild Acute Respiratory Distress Syndrome Patients: Preliminary Results

**DOI:** 10.2478/jccm-2024-0005

**Published:** 2024-01-30

**Authors:** Mihai Claudiu Pui, Mihaela Butiulca, Vlad Cehan, Florin Stoica, Alexandra Lazar

**Affiliations:** Department of Anesthesiology and Intensive Care Medicine, Emergency County Hospital, Targu Mures, Romania; Department of Anesthesiology and Intensive Care Medicine, Faculty of General Medicine, George Emil Palade University of Medicine, Pharmacy, Science, and Technology of Targu Mures, Romania; Department of Internal Medicine, Emergency County Hospital, Targu Mures, Romania; George Emil Palade University of Medicine, Pharmacy, Science, and Technology of Targu Mures, Romania

**Keywords:** acute respiratory distress syndrome, neutrophil-to-lymphocyte ratio, thrombocyte-to-lymphocyte ratio

## Abstract

**Introduction:**

Acute respiratory distress syndrome (ARDS) represents a major cause of mortality in the intensive care unit (ICU). The inflammatory response is escalated by the cytokines and chemokines released by neutrophils, therefore the search for quantifying the impact of this pathophysiological mechanism is imperative. Neutrophil/lymphocyte ratio (NLR) and platelet/lymphocyte ratio (PLR) are indicators of systemic inflammation, widely accessible, inexpensive, and uncomplicated parameters.

**Methods:**

We conducted a prospective study between March 2023 and June 2023 on patients which presented Berlin criteria for the diagnosis of ARDS during the first 24 hours from admission in the ICU. We included 33 patients who were divided into two groups: one group of 11 patients with severe ARDS and the second group of 22 patients with moderate/mild ARDS. The study evaluated demographic characteristics, leukocyte, lymphocyte, neutrophil, and platelet counts, as well as NLR and PLR values from complete blood count, and severity scores (APACHE II score and SOFA score). We investigated the correlation of NLR and PLR in the two main groups (severe and moderate/mild acute respiratory distress syndrome patients).

**Results:**

For the NLR ratio statistically significant differences between the two groups are noted: Severe ARDS 24.29(1.13–96) vs 15.67(1.69–49.71), p=0.02 For the PLR ratio, we obtained significant differences within the group presenting severe ARDS 470.3 (30.83–1427) vs. the group presenting mild/moderate ARDS 252.1 (0–1253). The difference between the two groups is statistically significant (0.049, p<0.05). The cut-off value of NLR resulted to be 23.64, with an Area Under the Curve (AUC) of 0.653 (95% CI: 0.43–0.88). The best cut-off value of PLR was performed to be 435.14, with an Area Under the Curve (AUC) of 0.645 (95% CI: 0.41–0.88).

**Conclusion:**

Our study showed that NLR and PLR ratios 24 hours in patients with moderate/severe ARDS diagnosis can be a good predictor for severity of the disease. These biomarkers could be used in clinical practice due to their convenience, inexpensiveness, and simplicity of parameters. However, further investigations with larger populations of ARDS patients are necessary to support and validate these current findings.

## Introduction

One of the major factors contributing to increased mortality in intensive care units (ICU) is acute respiratory distress syndrome (ARDS) [1]. It is widely established that inflammation influences the prognosis and symptoms of ARDS and plays a significant role in its progression. Neutrophil/lymphocyte ratio (NLR) and platelet/lymphocyte ratio are indicators of systemic inflammation. Elevated levels of neutrophil infiltration may be associated with cytotoxicity, vascular stasis, and decreased inflammation in response to changes in the balance of pro-inflammatory and anti-inflammatory cytokines [2,3]. The neutrophil to lymphocyte ratio (NLR), which is derived from neutrophil and lymphocyte values from a complete blood count (CBC), is thought to represent the degree of inflammation and can predict the progression and mortality rates of many inflammatory diseases. In inflammatory disorders, the platelet/lymphocyte ratio (PLR) is utilized to forecast the inflammatory phase, disease activity, responsiveness to treatment, and prognosis [4,5].

Neutrophil to lymphocyte ratio (NLR) serves, as a biomarker that establishes two stages of the immune system: chronic or acute inflammation (marked by the neutrophil component) as well as the capacity of immunity to adapt (supported by lymphocytes). Neutrophils trigger the first wave of an immune response aimed at various pathogens through an entire series of cellular mechanisms such as phagocytosis, chemotaxis, and releasing reactive oxygen species, cytokines, or granular proteins [6]. Regulating innate immunity, neutrophils establish the role of different cytokines with important roles in inflammation or immunomodulation such as NK cells, CD4, CD8, dendritic cells, or mesenchymal cells [7].

Firstly, PLR can expose the cytokine storm degree and can easily and inexpensively characterize the inflammatory state for ICU-admitted patients. The main benefit of PLR is that it informs the clinician about the aggregation pathway related to the inflammatory processes, research has yet to prove the capacity of prediction of specific inflammatory pathologies [8]. Several studies have established the relationship between the poor outcome and low lymphocyte values correlated with elevated platelet count for acute coronary syndrome. Furthermore, the link between PLR and death rate has been proven to be stronger than the independent effect of platelet or lymphocyte counts on mortality [9].

Previous studies have identified an association between neutrophil-to-lymphocyte ratio (NLR), platelet-to-lymphocyte ratio, and prognosis in critically ill patients [10]. This study investigated the prognostic and predictive value of NLR and PLR in patients with ARDS. The prognosis of critically ill patients has been determined using the Acute Physiology and Chronic Health Evaluation II (APACHE II) and the sequential organ failure assessment score (SOFA) scores.

However, most current biomarker studies require special biological samples of patients, a specific group of patients, and integration with other clinical data. Thus, the complexity and heterogeneity of the disease make this assessment very challenging [11,12].

Studies have shown a correlation between NLR, PLR, and severity of clinical course in patients in ICU suggesting that inflammatory index should be considered as a prognostic indicator. As prognostic indicators, NLR and PLR are far simpler, less expensive, and more readily available to use than other biomarkers, which can be quite challenging to deploy [13].

## Material and Method

### Design

We conducted a prospective study in the ICU department of the Emergency County Clinical Hospital in Târgu Mureș, Romania between March 2023 and June 2023. The study evaluated demographic characteristics, leukocyte, lymphocyte, neutrophil, and platelet counts, as well as NLR and PLR values from complete blood count, and severity scores (APACHE II and SOFA), all determined within the first 24 hours of admission to the ICU. Approval was obtained from the local ethics committee number 5453/15.03.2023.

### Study participants

The study included critically ill patients admitted to the ICU. Patients were over 18 years of age, tracheal intubated, and had ARDS within the first 24 hours from admission in the ICU. Patients who were younger than 18 years of age, those who were not tracheal intubated, those who had less than 24 hours of ICU admission, and patients who did not present ARDS criteria within the first 24 hours in the ICU were excluded. The final number included 33 patients who were divided into two groups: one group of 11 patients with severe ARDS and the second group of 22 patients with moderate/mild ARDS. Inclusion criteria for ARDS were bilateral infiltrates with edema on chest radiograph, PaO2/FiO2 less than 300, at least one risk factor for ARDS, tracheal intubation, and mechanical ventilation. Stratification for severe/moderate/mild ARDS was based on ventilation/perfusion ratio (P/F).

### Study objectives

The study’s main objectives were to assess the NLP and PLR correlation with ARDS severity, and their association with the severity scores (APACHE and SOFA 2) in ARDS patients.

### Statistical analysis

R Statistical Language (RStudio software) and Graph-Pad Prism 9 were used to perform the statistical analysis. Excel sheets were used to arrange and statistically evaluate the data. Continuous data are presented as medians (minimum-maximum) or means (± standard deviation), and categorical data as proportions. The Kolmogorov-Smirnov test was used to assess the normal distribution of continuous numerical variables. The Mann-Whitney U test was used for non-parametric variables, and the Student’s t-test was used for parametric continuous variables. Receiver operating characteristic (ROC) curve analysis was performed. Area Under the Curve (AUC) values were displayed, including a 95% confidence interval (CI). Spearman’s coefficients were used for correlations and a p-value less than 0.05 was considered statistically significant for the statistical analysis.

## Results

We analyzed 33 patients with ARDS who met the Berlin A criteria on admission, then created two groups based on the severity of ARDS. 11 patients were enrolled in the severe ARDS group and 22 patients were enrolled in the mild/moderate ARDS group.

The results obtained for NLR represent the median in the whole sample 16.90 (1.13–96), statistically significant differences between two groups are noted: Severe ARDS 24.29 (1.13–96) vs 15.67(1.69–49.71) p=0.02, Student’s t-test statistically significant result.

For the PLR ratio, we obtained a median for the whole population of 315.8 (0–1427), with statistically significant differences between the group presenting severe ARDS 470.3 (30.83–1427) vs. the group presenting mild/moderate ARDS. 252.1 (0–1253). The difference between the two groups is statistically significant (p=0.049), the test applied being Mann Whitney test.

The APACHE II severity score recorded mean value of 26.42±5.14, within the severe ARDS group 26.30±7.514, and for mild/medium ARDS 26.48±3.77. The p-value was p=0.93, obtained with Student’s t-test, with a statistically insignificant value (p>0.05). For the SOFA score we obtained a mean value of 10.33±3.22, within the severe ARDS group 10.55±3.446 and for mild/moderate ARDS the mean value is 10.23±3.19. The p-value obtained for the SOFA 2 score between the 2 groups was (p=0.79), after application of the Mann-Whitney test, not statistically significant.

In the whole population studied, statistically significant values (p<0.05) were observed for variables included in the blood gas analysis such as pO2 and HCO3. (Table 1)

**Table 1.** Baseline characteristics

	**All patients (n=33)**	**Severe ARDS (n=11)**	**Mild/Moderate ARDS (n=22)**	**P**
Age (years, mean±SD)	71.91±11.54	70.73±13.69	72.50±10.61	0.68*
GCS (median, min-max)	8(3–15)	6(3–15)	8(3–15)	0.69*
IOT-VM(n,%)	26(78.78)	9(81.81)	17(77.27)	0.76***
IOT-S(n,%)	1(3.03)	0(0)	1(4.54)	0.15***
NIV(n,%)	1(3.03)	0(0)	1(4.54)	0.15***
MF(n,%)	5(15.15)	2(18.18)	3(13.63)	0.73***
SpO2(%, mean±SD)	95.97±5.42	93.64±7.43	97.14±3.77	0.40**
FiO2(%, mean±SD)	68.94±20.61	76.36±23.78	65.23±18.29	0.16**
pH(mean±SD)	7.306±0.14	7.304±0.13	7.315±0.14	0.83*
pO2(mmHg, mean±SD)	91.68±40.42	59.61±15.27	107.7±39.63	<0.0001**
pCO2(mmHg, mean±SD)	50.65±20.50	46.05±16.47	52.95±22.25	0.44**
HCO3-(mEq/L, mean±SD)	25.16±6.35	22.12±5.28	26.68±6.40	0.0491**
Leukocytes (cells/µL, mean±SD)	15.91±7.50	16.52±5.66	15.60±8.38	0.7435*
Neutrophils (cells/µL, median, min-max)	10.93(3.47–84.70)	11.69(8.5–84.70)	10.69(3.47–40.45)	0.19**
Lymphocytes (cells/µL, median, min-max)	0.7(0.15–8.24)	0.6(0.15–8.24)	0.71(0.2–7.09)	0.55**
NLR(median, min-max)	16.90(1.13–96)	24.29(1.13–96)	15.67(1.69–49.71)	0.02*
PLR(median, min-max)	315.8(0–1427)	470.3(30.83–1427)	252.1(0–1253)	0.049**
APACHE II Score(mean±SD)	26.42±5.14	26.30±7.514	26.48±3.77	0.93*
SOFA (mean±SD)	10.33±3.22	10.55±3.446	10.23±3.19	0.79**
Outcome(n,%)	29(87.87)	10(90.90)	19(86.36)	0.70***

Abbreviations: ARDS-acute respiratory distress syndrome; NLR = Neutrophile Lymphocyte Ratio; PLR = Platelets Lymphocyte Ratio; APACHE II - The Acute Physiology and Chronic Health Evaluation; SOFA - The Sequential Organ Failure Assessment; GCS = Glasgow Coma Scale; MF= Facial mask; NIV= non invasive ventilation; IOT-S= tracheal intubation with T-piece; IOT-VM= tracheal intubation mechanical ventilated; SpO2=oxygen saturation; FiO2=inspiratory oxygen fraction; pO2=partial pressure of oxygen; pCO2=partial pressure of carbon dioxide; HCO3=bicarbonate;

* = Student test;

** = Mann Whitney test;

*** = Chi-square Test;

SD=Standard Deviation

**Fig. 1.**
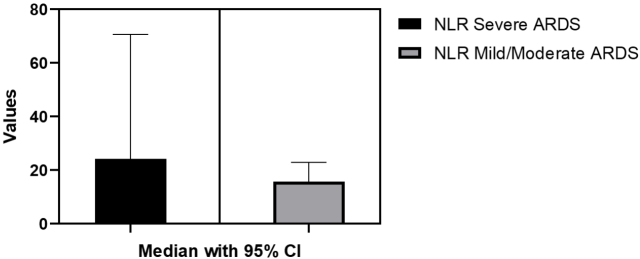
**NLP differences between Severe ARDS and Mild/Moderate ARDS.** NLR- Neutrophil-to-lymphocyte ratio; ARDS-acute respiratory distress syndrome; CI-confidence interval

Statistically significant differences were identified between the NLP values in the Severe ARDS vs Mild/Moderate ARDS groups after performing the Unpaired T test (p=0.025). Mean difference was 17.59±7.46 (Figure 1).

**Fig. 2.**
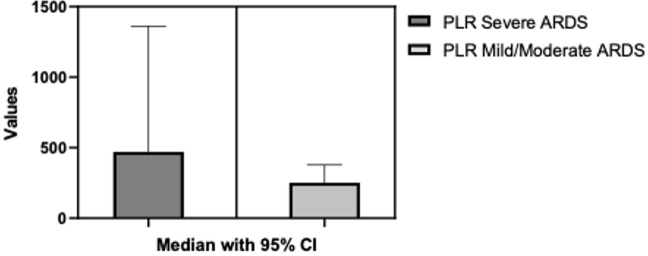
**PLR differences between Severe ARDS and Moderate/Milld ARDS.** PLR- Platelets-to-lymphocyte ratio; ARDS- acute respiratory distress syndrome; CI-confidence interval

No statistically significant differences were identified between the PLR values in the Severe ARDS vs Mild/Moderate ARDS groups following the Mann Whitney test (p=0.1908). The median of the first column was 252.1 (n=11) and the mean of the second column was 252.2 (n=22) (Figure 2).

**Fig. 3.**
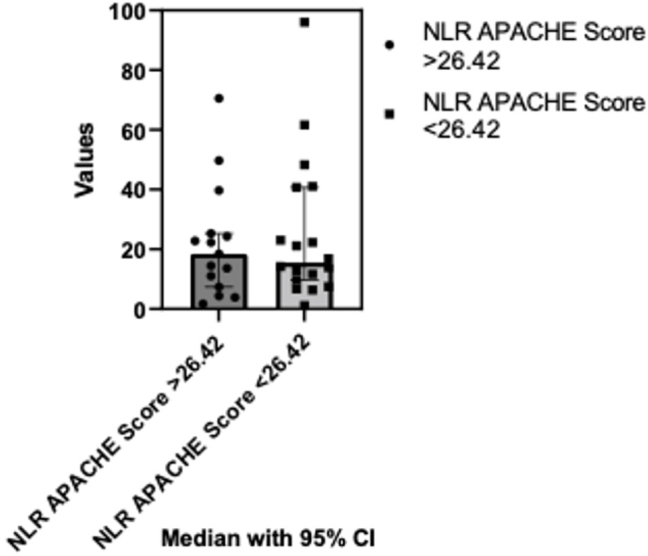
**Comparison of NLR variations based on APACHE II score: below and above mean score analysis.** NLR- Neutrophil-to-lymphocyte ratio; APACHE II - The Acute Physiology and Chronic Health Evaluation

In the comparison of NLR variations based on the APACHE Score from below and above mean (26.42), no statistically significant differences were identified following the Mann Whitney test (p=0.77). The median of the first column was 18.44 (n=15) and the median of the second column was 14.03 (n=16) (Figure 3).

**Fig. 4.**
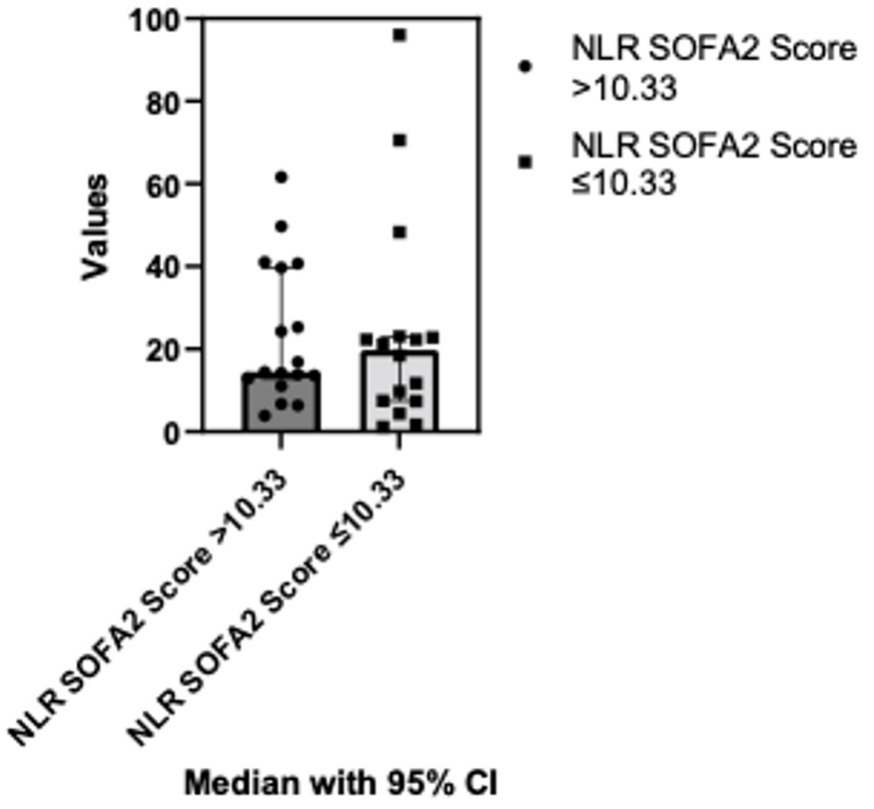
**Comparison of NLR variations based on SOFA2 score: below and above mean score analysis.** NLR- Neutrophil-to-lymphocyte ratio; SOFA - The Sequential Organ Failure Assessment; CI – Confidence interval

Comparing NLR variations based on SOFA Score from below and above mean (10.33) no statistically significant differences were found following the Mann Whitney test (p=0.65). The median of the first column was 14.44 (n=17) and the median of the second column was 19.79 (n=16) (Figure 4).

**Fig. 5.**
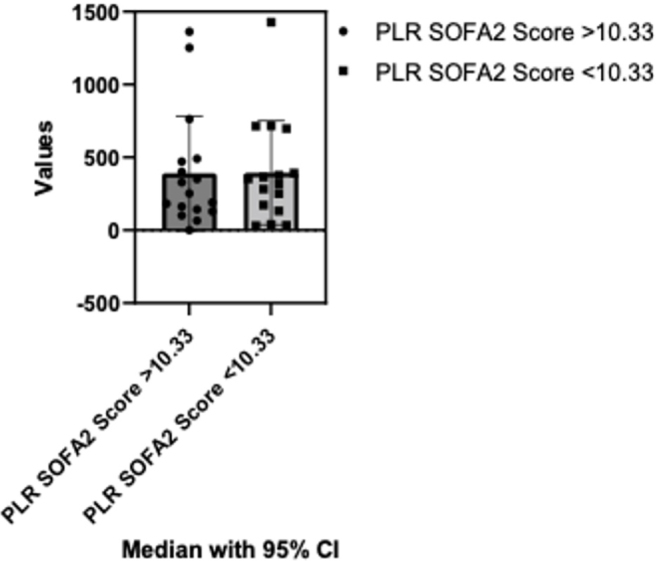
**Comparison of PLR variations based on SOFA score: below and above mean score analysis.** PLR- Platelets-to-lymphocyte ratio; SOFA - The Sequential Organ Failure Assessment; CI – Confidence interval

For the comparison of PLR variations based on SOFA Score from below and above the mean (10.33), no statistically significant differences were identified following the Mann Whitney test (p=0.84). The median of the first column was 252.8 (n=17) and the median of the second column was 333.9 (n=16) (Figure 5).

**Fig. 6.**
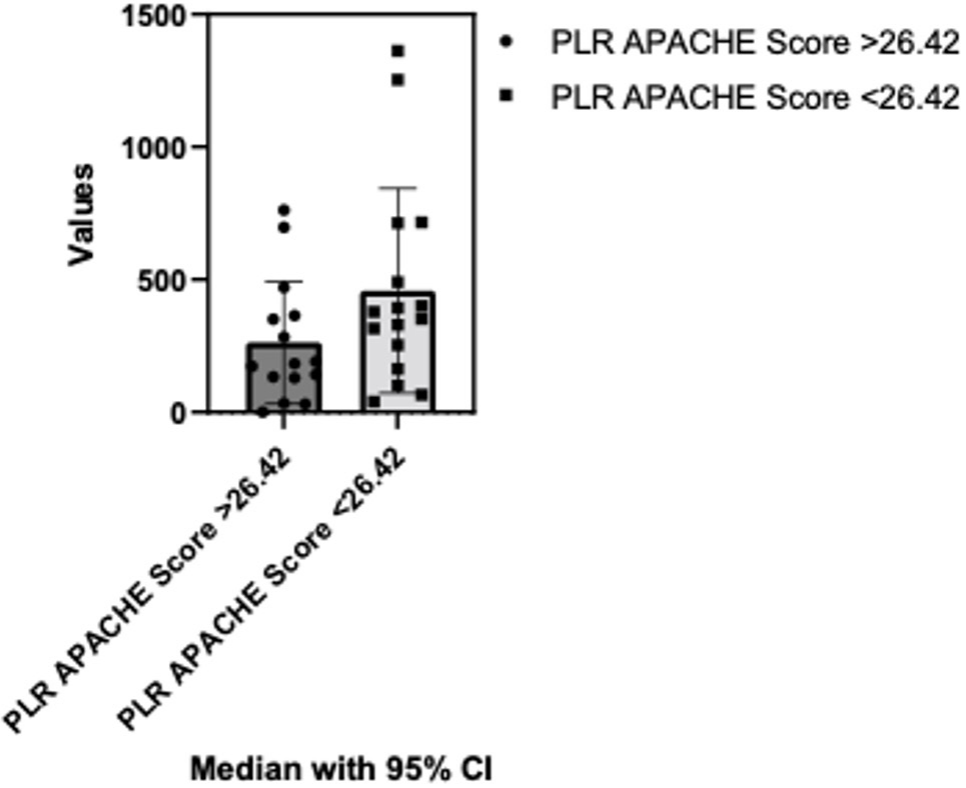
**Comparison between PLR variations based on APACHE II score: below and above mean score analysis.** PLR- Platelets-to-lymphocyte ratio; APACHE II - The Acute Physiology and Chronic Health Evaluation; CI – Confidence interval

While assessing PLR variations based on APACHE II Score comparing below and above the mean (26.42) values resulted in no statistically significant differences after applying the Mann Whitney test (p=0.10). The median of the first column was 182.5 (n=15) and the median of the second column was 364.9 (n=16) (Figure 6).

**Fig. 7.**
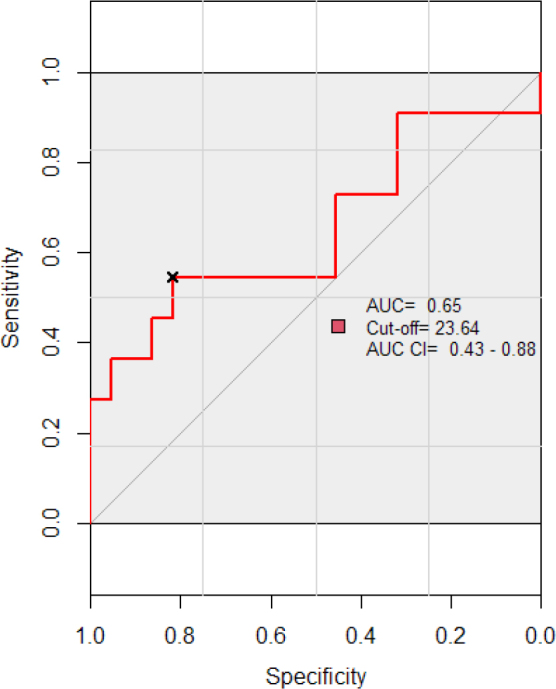
**Investigation of NLR cutoff value based on ARDS severity: ROC curve analysis.** ROC-Receiver Operating Characteristic; NLR- Neutrophil-to-lymphocyte ratio; AUC-Area Under the Curve

Receiver Operating Characteristic (ROC) curve analysis was performed to evaluate the diagnostic accuracy of NLR in differentiating between severe and mild/moderate ARDS. The cut-off value of NLR resulted to be 23.64, with an Area Under the Curve (AUC) of 0.653 (95%CI: 0.43–0.88) p=0.15. For the cut-off value, sensitivity was 0.54 and specificity was 0.81. These results indicate the fact that the NLR has a moderate ability to discriminate between severe and mild/moderate ARDS patients. Although the result is not statistically significant. (Figure 7)

**Fig. 8.**
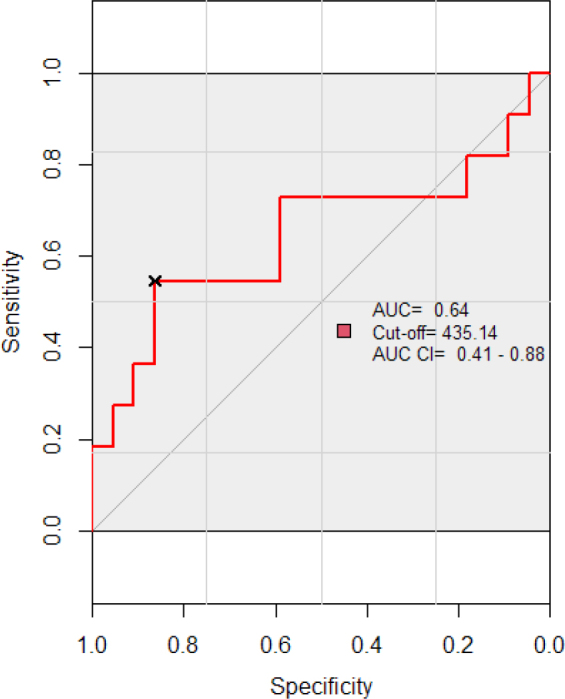
**Investigation of PLR cutoff value based on ARDS severity: ROC curve analysis.** ROC-Receiver Operating Characteristic; PLR- Platelets-tolymphocyte ratio; AUC-Area Under the Curve

Receiver Operating Characteristic (ROC) curve analysis was performed to evaluate the diagnostic accuracy of PLR in differentiating between severe and mild/moderate ARDS. The optimal cut-off value of NLR was performed to be 435.14, with an Area Under the Curve (AUC) of 0.645 (95%CI: 0.41–0.88), p=0.18. For the cut-off value sensitivity was 0.54 and specificity was 0.86. This indicates that the PLR has a moderate ability to discriminate between severe and mild/moderate ARDS patients. Although the result is not statistically significant. (Figure 8)

## Discussions

ARDS is an inflammatory reaction that affects the alveolar epithelium and pulmonary endothelium. Following this inflammation, capillary permeability increases, endothelial and epithelial cells are damaged, and ventilation-perfusion becomes inadequate, leading to alveolar edema, decreased lung expansion capacity, and persistent hypoxia, leading to, severe hypoxia and extensive bilateral infiltration on chest radiography, noncardiogenic pulmonary edema according with Berlin Criteria for ARDS [14,15].

NLR can predict progression and mortality rates in various inflammatory conditions. It’s association with the severity of inflammation is widely recognized. PLR is used to predict the duration of the inflammatory phase, disease activity, response to treatment and prognosis [16].

NLR and PLR values could be used to monitor the course of diseases dominated by systemic inflammation. NLR has been identified as a contributing factor to mortality and morbidity among ICU patients. One study reveals a correlation between elevated NLR levels in pneumonia patients admitted to intensive care units and their increased mortality rates [17]. Another study in adult patients with ARDS demonstrates a similar association between high NLR levels in critically ill people and their clinical outcomes. Another study of adult tuberculosis patients with ARDS suggests that NLR may serve as a predictor of mortality rates and ARDS development [18].

Aijia Ma et al. obtained results similar (in our study NLR was 0.65) to the sample ARDS patients NLR (AUC 0.71), while the AUC for PLR (in our study PLR was 0.645) had a slightly lower value (AUC 0.59). He also calculated the AUC for PCT (AUC 0.494) and CRP (AUC 0.625) in ARDS, his sample having included 81 patients [19]. Another study calculated the cutoff value for NLR at 20.34 ((specificity: 0.734, sensitivity: 0.7), with an AUC for APACHE II score of 0.672 (95% CI, 0.597–0.750, P < 0.001), similar to our result (our cut-off for NLR was 23.64). His calculations combine NLR, Berlin criteria. and APACHE II, resulting in a higher value of AUC (95% CI, 0.731–0.855, P < 0.001) [20].

When deciphering the results, it is important to consider the various limitations of this study. The primary limitations include the prospective design, limited duration of observation, and a relatively small sample size of patients. It should be noted that NLR and PLR can be influenced by several factors, such as obesity, HIV, stroke, acute trauma, medication usage, emotional stress, and underlying comorbidities, which can impact neutrophil and lymphocyte counts. To strengthen the findings, future studies should involve larger cohorts of patients to provide more comprehensive and reliable results [21].

## Conclusions

In conclusion, the study suggests that both NLR and PLR could be used as predictive markers for severe and mild/moderate ARDS. However, the AUC of NLR was higher, and the correlation between NLR and severe ARDS respectively mild/moderate ARDS was significantly stronger than that of PLR. Therefore, we conclude that NLR could be developed as a predictive marker for ARDS.
